# Analytical Electron Microscopy for Characterization of Fluid or Semi-Solid Multiphase Systems Containing Nanoparticulate Material

**DOI:** 10.3390/pharmaceutics5010115

**Published:** 2013-02-05

**Authors:** Victoria Klang, Claudia Valenta, Nadejda B. Matsko

**Affiliations:** 1 Research Platform Characterisation of Drug Delivery Systems on Skin and Investigation of Involved Mechanisms, University of Vienna, Althanstraße 14, 1090 Vienna, Austria; E-Mails: victoria.klang.vie@gmail.com (V.K.); claudia.valenta@univie.ac.at (C.V.); 2 Centre for Electron Microscopy Graz and Institute for Electron Microscopy and Fine Structure Research (FELMI-ZFE), Graz University of Technology, Steyrergasse 17, 8010 Graz, Austria

**Keywords:** nanoparticles, nanoemulsion, emulsion, analytical electron microscopy, energy-filtered transmission electron microscopy, energy-dispersive X-ray spectroscopy, electron energy loss spectroscopy

## Abstract

The analysis of nanomaterials in pharmaceutical or cosmetic preparations is an important aspect both in formulation development and quality control of marketed products. Despite the increased popularity of nanoparticulate compounds especially in dermal preparations such as emulsions, methods and protocols of analysis for the characterization of such systems are scarce. This work combines an original sample preparation procedure along with different methods of analytical electron microscopy for the comprehensive analysis of fluid or semi-solid dermal preparations containing nanoparticulate material. Energy-filtered transmission electron microscopy, energy-dispersive X-ray spectroscopy, electron energy loss spectroscopy and high resolution imaging were performed on model emulsions and a marketed product to reveal different structural aspects of both the emulsion bulk phase and incorporated nanosized material. An innovative analytical approach for the determination of the physical stability of the emulsion under investigation is presented. Advantages and limitations of the employed analytical imaging techniques are highlighted.

## Abbreviations

EELS: electron energy loss spectroscopy
EDXS: energy-dispersive X-ray spectroscopy
TEM: transmission electron microscopy
ATEM: analytical transmission electron microscopy
EFTEM: energy-filtered transmission electron microscopy
STEM: scanning transmission electron microscopy
ELNES: energy-loss near edge structure
PCR image: plasmon to carbon ratio image

## 1. Introduction

The technological and methodological advances in the field of pharmaceutical technology have led to an increasing interest in nanoparticulate drug delivery systems. Especially in dermal drug delivery, significant amounts of research are devoted to lipid-based colloidal carriers within the nanometer range, such as liposomes, nanoemulsions, solid lipid nanoparticles or polymeric nanoparticles [1]. These formulations are frequently adapted for cosmetic applications as well. 

Nanoparticles have been employed in certain topical systems such as sunscreens for a long time. The growing number of marketed products involving nanoparticles of various forms necessitates the development of suitable techniques of analysis to ensure consumer safety. Frequently, product claims are made in context with nanoparticulate material, and few valid options to investigate the presence or absence of nanoparticles in dermal preparations exist. The detection of solid nanoparticles among soft nanocarrier vehicles such as nanoemulsions is not easily feasible by standard techniques of particle size analysis such as dynamic light scattering. More importantly, the identity and morphology of the nanoparticles cannot be determined in this fashion. In this respect, electron microscopy may offer a powerful solution. 

Electron microscopic methods are increasingly employed for the characterization of pharmaceutical systems such as classical or lipid nanoparticles, nanoemulsions, microemulsions, nanofibers and many more [2]. Conventional and cryo transmission electron microscopic techniques frequently have to be adapted for an accurate analysis of formulation morphology, especially in case of hydrated colloidal systems. However, analytical electron microscopic techniques [3,4] such as electron energy loss spectroscopy (EELS) or energy-dispersive X-ray spectroscopy (EDXS), which have great potential to determine different structural and chemical aspects of both bulk phase and incorporated nanosized material [5,6,7,8,9,10], are not yet widely employed tools in pharmaceutical research. Thus, we would like to propose a working scheme that combines a suitable sample preparation approach and different analytical electron microscopic techniques for the comprehensive analysis of conventional pharmaceutical materials such as fluid or semi-solid dermal preparations containing nanoparticulate materials.

## 2. Materials and Methods

Previously developed emulsions of different droplet size and viscosity [11] as well as a fluid marketed emulsion product (Neosino^®^Spray Mild, Neosino, Austria) were investigated by conventional transmission electron microscopy (TEM) as well as analytical TEM (ATEM), which includes electron energy loss spectroscopy (EELS), energy-filtered TEM (EFTEM), and energy-dispersive X-ray spectroscopy (EDXS) in both TEM and scanning TEM (STEM) modes. 

Commercially available titanium dioxide nanoparticles (titanium(IV)oxide, average diameter <100 nm, CAS 13463677, Sigma Aldrich, St. Louis, MO, USA) were incorporated into the aqueous phase of plain emulsion systems by mechanical stirring and ultrasonication to improve their distribution within the systems. A concentration of 4% *w*/*w* was chosen since it is representative of commercial sunscreen formulations. 

For electron microscopic analysis, emulsions or other viscous liquids can be dissolved, e.g., in ethanol, to destroy and separate the emulsion matrix from the nanoparticles. Such sample preparation methods may change the properties of nanoparticles within the sample, such as their coating properties or aggregation tendencies. Thus, the presented analyses were performed without prior sample treatment. A small droplet of the initial formulations was placed onto plastic paraffin films and covered by a holey carbon coated copper EM grid for a few seconds. Afterwards, the grids were gently polished with filter paper to remove excess material and then left to dry. 

In order to gain electron energy loss (EELS) reference spectra of titanium and carbon, the nanoparticles were dissolved in distilled water, ultrasonicated and immediately applied to the holy carbon coated copper grids as described above.

All TEM analyses were performed on a Philips CM 20 (equipped with LaB_6_ cathode) and Tecnai TF20 FEG (FEI, Eindhoven, The Netherlands) electron microscopes at 200 kV accelerating voltage. The spectra and images were recorded with slow-scan CCD camera integrated in the GIF (YAG scintillator crystal; 1024 × 1024 pixel array). For recording of bright field TEM images 1024 × 1024 pixels were used while energy-filtered images at any energy loss region were recorded using a binning of 2 × 2 thus resulting in 512 × 512 pixels. Since the optimal defocus for an energy filtered image differs significantly from that for an elastic image, the focus has been adjusted in an energy filtered image, obtained at about 100 eV energy loss, by using the real-time CCD camera of the GIF before recording the energy-filtered images with the slow-scan CCD camera. All EFTEM and EELS experiments were acquired in TEM mode. Experimental conditions for the acquisition of elemental maps: energy slit width 30 eV, acquisition time 20 s for Ti L ionization edge (481 eV), energy slit width 10 eV, acquisition time 10 s for Si L ionization edge (109 eV), and energy slit width 30 eV, acquisition time 30 s for O K ionization edge (547 eV). For the calculation of plasmon to carbon ratio image (PCR image) [12], all sets of energy filtered images (C K elemental map, and valence bulk plasmon) have been carefully drift corrected using cross-correlation algorithm developed by Schaffer and colleagues [13]. Energy-filtered bulk plasmon images were recorded at 25 eV with an energy window width of 10 eV and the C elemental map with a slit width of 16 eV at 292 eV. All data processing has been performed using the Digital Micrograph software (Gatan, Pleasanton, CA, USA). The negative staining of the samples with uranyl acetate was performed as reported [14].

All EDXS experiments were performed in STEM mode with a probe current of 20 nA and a beam diameter of 100 nm. EDXS spectra were collected using a high-purity germanium detector (HPGe detector, solid angle 0.13 sr). 

All EDXS spectra, EFTEM elemental maps of Ti, O, and Si as well as the EELS spectra were recorded without prior staining. 

## 3. Results and Discussion

The main requirement for a successful TEM analysis is the formation of a film that is thin enough to transmit a sufficiently large number of electrons, so enough intensity falls on the CCD to give an interpretable image within a reasonable time frame. In extreme cases, e.g., during high resolution TEM analysis or electron spectrometry, specimen thicknesses less than 50 nm or even less than 10 nm are required [3,4]. There are different techniques to produce TEM specimens from hydrated pharmaceutical systems such as emulsions. For analysis at room temperature, such samples are placed on a grid covered with a relatively stable supportive film of carbon or formvar with a thickness of 20 to 100 nm [2,11]. For conventional TEM analysis, this additional film does not represent a limitation since carbon-based materials are light elements that scatter electrons rather weakly. Thus, organic samples with a thickness of up to 200 nm can still be used for TEM analysis even at relatively low voltages (60–120 eV) [4].

However, high resolution imaging and spectrometry require much thinner specimens and additional supportive films have to be avoided since the involved side elements may bias the results of the original formulation. At cryo conditions, this goal is achieved by freezing a thin sample layer located within the holes of a holey carbon grid. Since the structural organisation of an emulsion is preserved by vitrification, such samples remain solid at vacuum conditions within the TEM [15]. From our experience, liquid emulsions including nanoemulsions can be transferred to the holes of a holey carbon film at room temperature as well. Due to the high surface tension observed for the presented emulsion systems, a very thin sample layer is formed during removal of excess sample with filter paper. This sample layer is stable enough to be dried and be observed by TEM without any additional sample treatment. Such a sample preparation procedure is very helpful when the goal is high resolution investigation and also analytical TEM. 

In this study, both TEM and ATEM were performed to obtain a comprehensive chemical description of both the model emulsions and a marketed product. Each of the proposed techniques provides complementary information about formulation properties and the properties of the involved nano-sized material. It is thus possible to obtain information about nanoparticulate material in hydrated samples, the elemental composition as well as topographic information after dehydration of the samples. 

**Figure 1:**
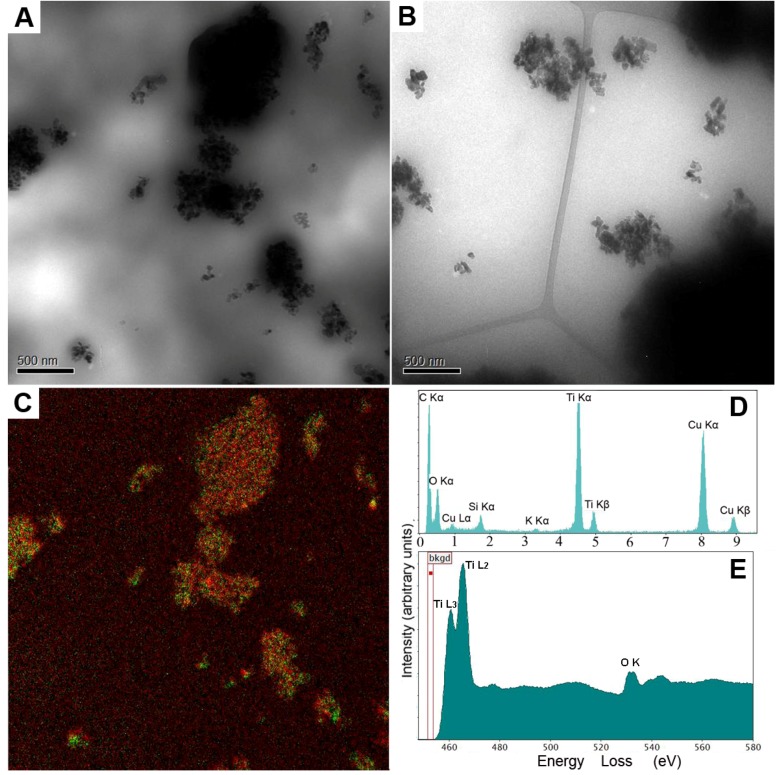
Analytical transmission electron microscopy (ATEM) characterisation of the model macroemulsion (**A** as well as **C**, **D** and **E**) and nanoemulsion (**B**) with incorporated titanium dioxide particles by energy-filtered transmission electron microscopy (EFTEM), energy-dispersive X-ray spectroscopy (EDXS) and electron energy loss spectroscopy (EELS) analyses without prior staining. Images (**A**) and (**B**) show zero-loss filtered bright field TEM images of the macroemulsion (**A**) and the nanoemulsion (**B**) (scale bars represent 500 nm). Image (**C**) shows a representative multicoloured energy-filtered image of superimposed titanium (Ti L) and oxygen (O K) maps, *i.e.*, the elemental distribution, obtained from the macroemulsion sample. Titanium is marked in green while oxygen is marked in red. Image (**D**) shows an EDXS spectrum of the macroemulsion. Image (**E**) represents an EELS spectrum obtained from a titanium dioxide aggregate within the macroemulsion.

Information about the sample composition was obtained by EELS, EFTEM, and EDXS. Figure 1 shows the results obtained for the model macroemulsion and nanoemulsion. By conventional TEM, information about the presence of nanoparticles within the samples was obtained. As can be seen in Figure 1A,B, large amounts of nanoparticle aggregates were observed. Figure 1C shows a representative multicoloured energy-filtered image of superimposed titanium and oxygen maps obtained from the macroemulsion sample. Titanium is marked in green, oxygen is marked in red. Comparative results were obtained for the nanoemulsion (data not shown). The technique of EFTEM can be employed to identify and localize nanoparticles, even if particles of different chemical composition are present. It should be noted that dehydrated samples may be partially damaged and/or drifting during image acquisition in EFTEM. Thus, slight deviations in the color map may be observed regarding the location of titanium and oxygen. An absolutely accurate fit as for solid samples can be obtained by means of more elaborate sample preparation, e.g., by separating and drying of the nanoparticles.

In summary, the elemental distribution of the incorporated model nanoparticles in both the nanoemulsion and the macroemulsion could be visualized by EFTEM in form of a two-dimensional map. The distribution of titanium dioxide was rather similar for the macroemulsion and the nanoemulsion. A slightly more homogeneous nanoparticle distribution was observed for the macroemulsion, which is in accordance with the higher viscosity of this system that diminishes direct gravitational influences on the particles.

By EDXS, the full set of chemical elements that were present in the investigated formulations in concentrations above the detection limit was identified. The energy resolution that serves to identify individual elements by their peaks strongly depends on the detector collection angle and experimental conditions such as the employed voltage and brightness [4]. The current limitation of this technique lies within the range of 0.1% to 1% (*w*/*w*) of an individual element. Figure 1D shows a representative EDXS spectrum of the macroemulsion. The elements C, Ti, O, Si and K can be easily identified. 

**Figure 2:**
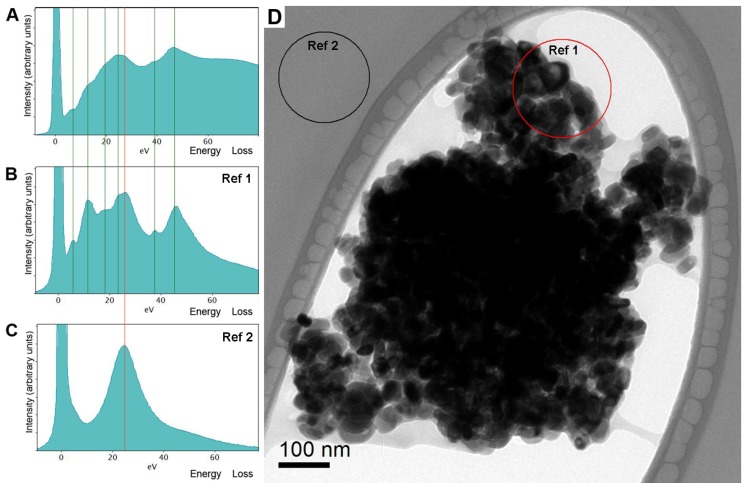
EELS spectra of bulk plasmon energy region obtained from the original macroemulsion (**A**) and titanium dioxide-carbon reference samples (**B**, **C**); The green lines show correlation between Ti low loss peaks which are present in both: low loss regions of macroemulsion and the reference spectra; red line indicates carbon low loss peak; (**D**) zero-loss filtered bright field TEM images of titanium dioxide-carbon reference sample. Selected areas indicate positions where EELS spectra for Ref. 1 and Ref. 2 were taken.

In addition, the chemical state of the detected elements was determined by EELS. An exemplary EELS spectrum is shown in Figure 2A, which represents a low loss region of an EELS spectrum obtained from a titanium dioxide aggregate. In case of our model formulations, well-separated plasmon peaks could be identified as superimposed signals from titanium dioxide (see reference spectrum 2B) and amorphous carbon (see reference specter 2C). The energy resolution of the spectrometer is determined by the electron source, which is approximately 1.2 eV (full width at half maximum of the zero-loss peak) in the current study. However, it has to be mentioned that due to the plural-scattering contribution to the background, the signal to background ratio falls rapidly as the specimen thickness (t) approaches the mean-free path (λ) for outer-shell inelastic scattering. For carbon, λ is approximately 160 nm (at 200 kV and the collection angle β near 7,6 mrad). The signal to noise ratio decreases after passing a certain thickness maximum as well. Therefore, an accurate quantitative elemental analysis cannot be performed for thick samples (t/λ over 1) [4].

**Figure 3:**
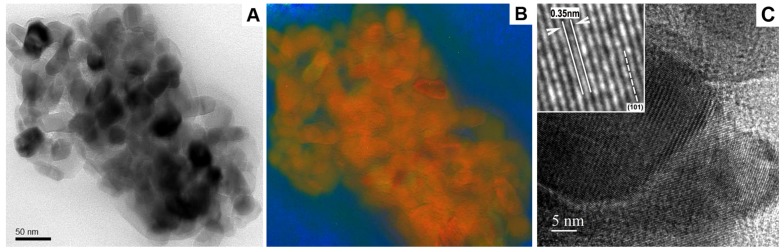
Characterization of the model macroemulsion with incorporated titanium dioxide particles by conventional zero-loss filtered bright field TEM (**A**) and EFTEM (**B**); The RGB EFTEM image shows the superimposed elemental maps of carbon (blue), titanium (red) and oxygen (green); (**C**) High resolution bright field TEM image of titanium dioxide particles. The scale bar represents 50 nm in (**A**, **B**) and 5 nm in (**C**).

Using high magnification, information about the precise morphology, elemental composition of individual particles and its atomic structure within the emulsion samples were obtained (Figure 3). The resolution limit of EFTEM mapping for routine investigations of soft materials, which usually have a strong drifting tendency during TEM investigations, is currently in the range of a few nanometers [4]. High resolution bright field imaging can be used where the purpose is to maximize useful details concerning the nanoparticles, such as the type of crystal polymorph, nanoporosity or specific surface area of functionalized nanoparticles with modified surface structures. In this respect, Figure 3C clearly indicates that the TiO_2_ nanocrystal lattice fringes are spaced at 0.35 nm and can be assigned to the (101) type planes [16]. 

It has to be mentioned that the use of ATEM imaging at cryo conditions in this particular case is strongly limited, especially at high resolution. Beam damage occurs, especially since cryo ATEM usually requires the use of primary energies of 200 kV and rather long acquisition time, in contrast to the conventionally used 80–120 kV and short acquisition for cryo TEM imaging. The high vacuum conditions within the microscope column represent another limitation. All frozen hydrated systems are prone to electron beam damage, which results in unfavorable structural reorganization and a partial or even complete loss of native sample features during observation. This effect also significantly increases the sample drift during TEM analysis, which renders high resolution analysis impossible. The intensive sublimation and recrystallization of the ice within the frozen hydrated samples also leads to increasing statistical noise that obscures the weak energy-loss near edge structure (ELNES) signals, causing increased errors in background extrapolation and further deterioration of the detection limits [11]. Therefore, it is reasonable to employ ATEM to analyze dehydrated pharmaceutical systems—where possible—at room temperature to obtain high resolution analytical images. 

By means of EFTEM, the chemical composition of the observed nanoparticles and the localization of the different elements within the sample volume were determined in a 2D projection. In addition, an analytical PCR method [12] was employed to obtain information about the topographical surface profile and thus the mechanical properties of the interfacial film within the emulsion, in particular regarding its resistance to high vacuum and fast dehydration. During negative staining, heavy metal salts mainly cover the surface features of the sample. This is in contrast to other staining procedures, where uranyl acetate cations may chemically react with sample constituents and penetrate deep into the specimen volume [17,18,19,20]. Thus, negative staining allows a thin layer of uranyl acetate to serve as an analogue to a fine and precise metal replica of the sample surface. The heavy metal shadowing effect, which enhances the image contrast, is achieved due to the different amounts of the staining solution, which remains in the topographically highest and lowest areas of the sample surface after negative staining [19,20].

For negatively stained organic materials such as emulsions, the bulk plasmon loss energy filtering image [21] mostly represents carbon as chief element as well as the employed staining metals such as uranium. In this case, the C map is a conventional three-windows EFTEM map showing only the distribution of carbon atoms while the background has been removed [4]. The resulting plasmon to carbon ratio image therefore shows a fine distribution of uranium atoms, which replicate the sample surface topography with nanometer precision. In contrast to conventional EFTEM mapping (including an absolute quantification approach) [22,23], the proposed PCR method offers the advantage of eliminating thickness variation effects, which are especially pronounced for thick samples with a t over lambda—ratio higher than 0.9 [3,4,12,24].

**Figure 4:**
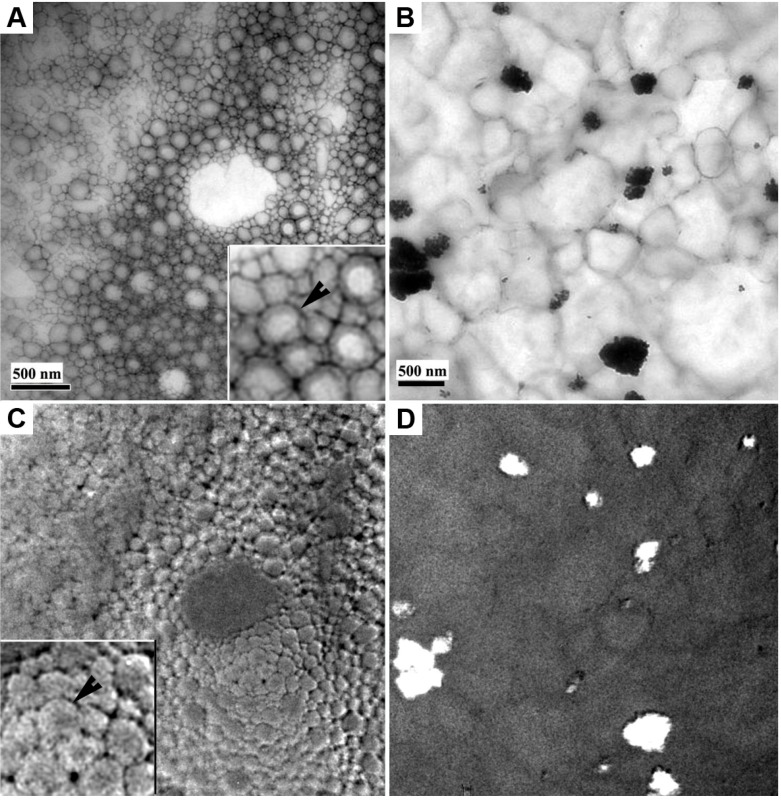
Conventional two-dimensional TEM projection images of the model nanoemulsion and macroemulsion with incorporated titanium dioxide particles and corresponding surface profiles as mathematically extracted from the EFTEM data. Both samples were negatively stained with a 2% aqueous uranyl acetate solution. Images (**A**) and (**B**) show zero-loss filtered bight field TEM images of the nanoemulsion (**A**) and the macroemulsion (**B**). The images below show the corresponding surface profile for the nanoemulsion (**C**) and for the macroemulsion (**D**) Black arrows indicate a hollow droplet shells composed of dried surfactant film. Scale bars represent 500 nm.

**Figure 5:**
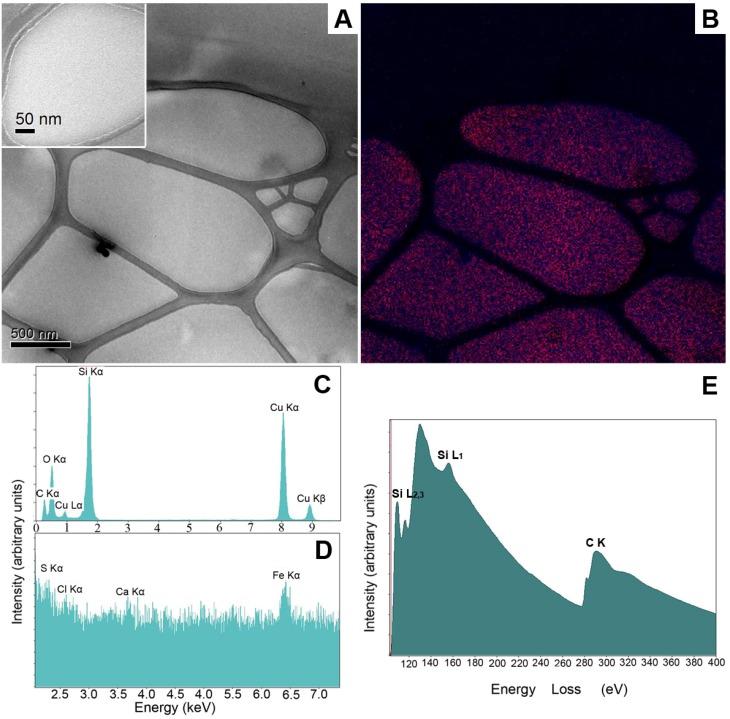
ATEM characterization of a commercially available liquid formulation containing unspecified nanomaterial by EFTEM, EDXS and EELS analyses without prior staining. Image (**A**) shows a zero-loss filtered bright field image of the formulation (scale bar represents 500 nm). In image (**B**), a multicolored EFTEM image of the elemental distribution within the formulation is given. The elemental distribution of silicon (blue) and oxygen (red) is documented in different colors. Images (**C**) and (**D**) show EDXS spectra of the same formulation with two different energy scales. In image (**E**), an EELS spectrum of the silicon (Si L) and carbon (C K) ionization edges are given.

In case of the nanoemulsion (Figure 4A), hollow droplet shells composed of dried surfactant film were clearly visible. In case of the macroemulsion (Figure 4B), only weak remnants of the original emulsion morphology could be distinguished. It may be assumed that the extent of preservation of the emulsion structure is directly connected to its physical stability. Thus, this technique allows for an estimation of the mechanical stability of the interfacial film within the system. Emulsions are metastable soft carriers and their shelf life strongly depends on the ability of the employed surfactant mixture to stabilize the emulsion droplets. When subjected to mechanical stress such as drying under high vacuum and electron irradiation within the electron microscope, the extent of preservation of the surfactant shells may serve to compare the stability of different emulsion systems. As can be seen, the nanoemulsion exhibited a marked surface profile (Figure 5C): individual droplet shells of former emulsified oil droplets could be distinguished. In contrast, the macroemulsion exhibited a relatively flat topography (Figure 4D); no individual droplet remnants could be seen. These observations correspond well with the data obtained by other methods of stability testing for emulsions, such as dynamic light scattering and laser diffraction [14]. The nanoemulsions exhibit superior physical stability, which is well reflected by the presented EM technique. Whether this method can serve to provide accurate stability information about emulsion systems will be subject of further investigations. 

Having adapted the proposed techniques for investigation of the model emulsions, a marketed fluid skin care lotion (Neosino^®^ Spray Mild, Neosino, Austria) with a product claim for “nanominerals” was analyzed (Figure 5). In this case, no nanoparticles were detected by TEM analysis (Figure 5A and 5A insert). The EFTEM maps of Si and O showed a comparatively homogeneous distribution of silica throughout the fluid system, as can be seen in Figure 5B. The EDXS spectra likewise supported this observation. The X-ray spectra taken of large as well as small specimen areas clearly indicated that Si was distributed homogeneously all over the sample volume and was not organized in clusters. In addition, fluctuations of small intensity from Ca, Cl and S were present in the EDXS spectra, but could not be clearly identified as peaks. These fluctuations may correspond to the lowest concentration that is detectable using the HPGe detector [4].

The EELS analysis confirmed the presence of large amounts of hydrated silica, apparently in form of a colloidal dispersion or solution without larger nanoparticulate aggregates. This suggests that the system indeed contained silica as given in the list of ingredients. The colloidal dispersion of silica may of course be referred to as “nano-material” as given in the product claim, although this may be misleading for consumers who are expecting specifically designed nanoparticles. 

## 4. Conclusions

The combination of the proposed ATEM techniques (EELS, EFTEM, EDXS and HRTEM) represents a powerful asset towards a substantiated structural characterization of nanoparticulate material in semi-solid or fluid emulsion systems. Open questions regarding the proposed analytical scheme concern the detection limits of the proposed ATEM methods. EDXS analysis should always be performed in combination with other imaging techniques for elemental and chemical mapping of the sample since the detected elements may of course not necessarily be present in nanoparticulate form.
